# Patient-derived heavy chain antibody targets cell surface HSP90 on breast tumors

**DOI:** 10.1186/s12885-015-1608-z

**Published:** 2015-09-03

**Authors:** Charan V. Devarakonda, Daniel Kita, Kathryn N. Phoenix, Kevin P. Claffey

**Affiliations:** Department of Cell Biology, Center for Vascular Biology, University of Connecticut Health Center, 263 Farmington Avenue, Lab E5029, Farmington, CT-06030-3501 USA

## Abstract

**Background:**

Monoclonal antibodies have been used to effectively treat various tumors. We previously established a unique strategy to identify tumor specific antibodies by capturing B-cell response against breast tumor antigens from patient-derived sentinel lymph nodes. Initial application of this approach led to identification of a tumor specific single domain antibody. In this paper we optimized our previous strategy by generating heavy chain antibodies (HCAbs) to overcome the deficiencies of single domain antibodies. Here we identified and characterized a heavy chain antibody (HCAb2) that targets cell surface HSP90 antigen on breast tumor cells but not normal cells.

**Methods:**

Eight HCAbs derived from 4 breast cancer patients were generated using an *in vitro* expression system. HCAbs were screened against normal breast cells (MCF10A, HMEC) and tumor cell lines (MCF7, MDA-MB-231) to identify cell surface targeting and tumor specific antibodies using flow cytometry and immunofluorescence. Results observed with cell lines were validated by screening a cohort of primary human breast normal and tumor tissues using immunofluorescence. Respective antigens for two HCAbs (HCAb1 and HCAb2) were identified using immunoprecipitation followed by mass spectrometry. Finally, we generated MDA-MB-231 xenograft tumors in NOD *scid* gamma mice and performed *in vivo* tumor targeting analysis of HCAb1 and HCAb2.

**Results:**

Flow cytometry screen revealed that HCAb2 selectively bound to the surface of MDA-MB-231 cells in comparison to MCF10A and MCF7 cells. HCAb2 showed punctate membrane staining on MDA-MB-231 cells and preferential binding to human breast tumor tissues in comparison to normal breast tissues. In primary breast tumor tissues, HCAb2 showed positive binding to both E-cadherin positive and negative tumor cells. We identified and validated the target antigen of HCAb2 as Heat shock protein 90 (HSP90). HCAb2 also selectively targeted MDA-MB-231 xenograft tumor cells *in vivo* with little targeting to mouse normal tissues. Finally, HCAb2 specifically targeted calnexin negative xenograft tumor cells.

**Conclusions:**

From our screening methodology, we identified HCAb2 as a breast tumor specific heavy chain antibody targeting cell surface HSP90. HCAb2 also targeted MDA-MB-231 tumor cells *in vivo* suggesting that HCAb2 could be an ideal tumor targeting antibody.

**Electronic supplementary material:**

The online version of this article (doi:10.1186/s12885-015-1608-z) contains supplementary material, which is available to authorized users.

## Background

Antibodies against various tumor associated antigens have been widely used in the treatment of different tumors [1–3]. Emergence of Cetuximab [4], Trastuzumab [5] and Ipilimumab [6] against solid tumors as well as Rituximab [7] and Ofatumubab [8] against hematological malignancies has highlighted the significant role and efficacy of antibodies in cancer therapy. Trastuzumab and Pertuzumab that target human epidermal growth factor receptor 2 (HER2) have been shown to synergestically inihibit growth of HER2 over-expressing breast cancer cells and also kill them [9]. These examples highlight the importance of antibodies in treatment of tumors as well as the need for identifying additional tumor specifc antibodies.

In order to develop tumor specific antibodies, identity of the target antigens has to be known. Previously described examples of tumor specific antibodies were developed by understanding the basic aspects of tumor biology. For instance, breast tumors that over-express HER2 receptor rely on this signaling pathway for survival and proliferation. Therefore, anti-HER2 receptor antibodies such as Trastuzumab and Pertuzumab were developed to specifically target HER2 over-expressing tumors. This targeted approach is highly successful but is limited by our understanding of tumor biology. Also this approach does not lead to identification of novel tumor associated antigens. Therefore, a strategy leading to the identification of novel tumor associated antigens as well as antibodies that target these antigens is warranted.

Humoral immune responses against tumor antigens have been observed in various cancer patients as evidenced by serum antibodies [10, 11] as well as activated B-cells in sentinel lymph nodes [12]. In our previous study, we established a unique strategy to identify novel tumor associated antigens [12]. Our strategy involved identification of activated and proliferating B-cells in sentinel lymph nodes of breast cancer patients. We hypothesized that these B-cells could have been activated by unique antigens derived from the tumors. Therefore, analyzing antibodies produced by these B-cells could lead to identification of tumor-associated antigens. Previously, we generated cDNA molecules of variable heavy chain domains from activated B-cells. Variable heavy chain cDNA molecules were sequenced and those that were part of clonal groups as well as exhibited somatic hypermutation within complementarity determining regions were selected for subsequent analysis. In our index study, single domain antibodies from activated B-cells were synthesized and screened to identify tumor-associated antigens [12]. Using this approach, neuroplastin was identified as a breast tumor associated antigen that was expressed at high levels in 20 % of invasive breast tumors and 50 % of those that became metastatic to distal sites. Identification of neuroplastin using these single domain antibodies validated the power of this research strategy to identify novel tumor antigens.

Single domain antibodies are small molecules (12-15 kDa) that can bind to antigens with similar affinity as intact antibodies [13–16]. But single domain antibodies lack Fc region and thereby cannot mediate effector functions such as antibody-dependent cell-mediated cytotoxicity (ADCC) and complement-dependent cytotoxicity (CDC). Also due to their small size, single domain antibodies have a rather short serum half-life [17] thereby requiring higher dosage for effective *in vivo* tumor targeting. In order to circumvent these problems, single domain heavy chain cDNAs were subcloned into a mammalian expression vector and heavy chain antibodies (HCAbs) made up of variable heavy chain regions fused to mouse Fc region were generated.

In this study, eight unique HCAbs (HCAb1-8) derived from our patient sentinel lymph node libraries were screened to identify potential tumor targeting antibodies. Of the 8 HCAbs, HCAb2 demonstrated preferential cell surface staining on MDA-MB-231 cells but not on normal cells and also selectively bound to human breast tumor tissues in comparison to normal tissues. The antigen for HCAb2 was identified to be cell surface HSP90 and consistent with HSP90 literature, HCAb2 reduced migration of MDA-MB-231 cells in *in vitro* migration assays. Finally, we showed that HCAb2 could target MDA-MB-231 tumor cells in an *in vivo* mouse xenograft model, thus defining a potentially useful anti-tumor antibody.

## Methods

### Patient samples

Primary breast normal tissues, tumor tissues and medial sections of lymph nodes were obtained soon after resection. Samples were placed in optimal cutting temperature (OCT) media and stored at -80 °C until further use. All participants provided written informed consent to participate in the study. All samples were collected in accordance with an IRB protocol (IRB number IE-01-205-2) approved by the Institutional Review Board at University of Connecticut Health Center and were devoid of any personal identification information.

### Cloning, synthesis and purification of heavy chain antibodies

Forty six different variable heavy chain clones were selected from our previously established cDNA libraries [12]. Variable heavy chain clones were sequenced and analyzed using IMGT/V-QUEST (http://www.imgt.org/IMGT_vquest/vquest) to determine V, D and J gene segment usage. Mutations within complementarity determining regions (CDRs 1, 2 and 3) as well as framework regions (FRs 1, 2, 3 and 4) were determined for each of the sequences in comparison to their respective germline sequences. Variable heavy chain sequences were subcloned from pCR®T7/CT-TOPO® (Life Technologies, NY, USA) plasmid into a mammalian expression vector pCMV6-AC-FC-S containing C-terminal mouse Fc sequence (OriGene technologies, MD, USA) using the following strategy. Variable heavy chain sequences were amplified by two rounds of PCR. First round of PCR was performed using forward primer 5ˈ- TTCGGCGATCGCCATGCAGGTGCAGCTGGTGSAGTCTGG - 3ˈ and reverse primer 5ˈ- GCCTTGGAAGTACAGGTTCTCACCGGTACGCGTAGAATCGAGACCGAG - 3ˈ, while second round of PCR was performed using the same forward primer and the following reverse primer 5ˈ- TGGGCTCGAGGCCTTGGAAGTACAGGTTCTCACCGGTACGCG - 3ˈ. PCR products were purified using QIAquick PCR purification kit (QIAGEN, CA, USA) according to manufacturer’s instructions. Purified PCR products and pCMV6-AC-FC-S plasmid were digested with AsiSI and XhoI (New England Biolabs, MA, USA) restriction enzymes for 1 h at 37 **°**C. PCR products were ligated with cut pCMV6-AC-FC-S plasmid and resultant transformants were screened by restriction digestion analysis. For this study, 8 variable heavy chain domain clones derived from four different breast cancer patient lymph node libraries were selected.

Purified plasmids containing variable heavy chain sequences were transfected into HEK293T cells. After 12-16 h, cells were washed with PBS and refed with serum free media. Heavy chain antibodies synthesized by HEK293T cells were released into conditioned media due to the presence of an N-terminal secretion signal sequence. 48 h after refeeding, conditioned media was collected and centrifuged at 300 g to pellet dead cells. Supernatants were filtered through 0.22 μm filter (polyethersulfone membrane, Millipore, MA, USA) and mixed with equal volume of PBS. Heavy chain antibodies were purified by protein A affinity chromatography using a 1 mL cartridge connected to an AKTA Purifier 10 system. Heavy chain antibodies were eluted with low pH glycine buffer (pH = 2.5) and neutralized with Tris buffer (pH = 8.0). Purified heavy chain antibodies were then concentrated and buffer exchanged using centrifugal concentrators (Microcon YM-30, Millipore). Absorbance of heavy chain antibodies was read at 280 nm and total protein content was determined using calculated extinction coefficients for each of the individual HCAbs [18].

### Cells and cell culture

All the different cells used in this study were obtained from American Type Culture Collection (ATCC, VA, USA). HMEC and MCF10A cells were cultured in mammary epithelial cell growth medium (MEGM - Lonza) supplemented with 50 units/mL of penicillin and 50 μg/mL of streptomycin, while HEK293T, MCF7 and MDA-MB-231 cells were cultured in Dulbecco’s modified Eagle’s medium (DMEM - Invitrogen) supplemented with 10 % fetal bovine serum, 50 units/mL of penicillin and 50 μg/mL of streptomycin.

### Antibodies

Antibodies against HSP90 (4877), clathrin heavy chain (4796), calnexin (2679) and cleaved caspase-3 (9661) were purchased from Cell Signaling Technology, MA, USA. Antibodies against HSP90 (sc-1055 and sc-1057) and E-cadherin (sc-7870) were purchased from Santa Cruz Biotechnology, TX, USA. Antibodies against CD31 (553370) and CD44-FITC (555478) were purchased from BD Biosciences, CA, USA. Alexa Fluor® 488 anti-mouse (A11001), Alexa Fluor® 594 anti-mouse (A11005), Alexa Fluor® 594 anti-rabbit (A11012) and Alexa Fluor® 594 anti-rat (A21209) antibodies were purchased from Life technologies, NY, USA.

### Flow cytometry

Cells were trypsinized with 0.05 % trypsin-EDTA (Life Technologies) and 0.5 × 10^6^ cells for each cell type were used for each analysis. Cells were washed thrice with Hank’s balanced salt solution containing 3 % fetal bovine serum and 1 mM EDTA (FACS buffer) and incubated with 10 μg of respective HCAbs for 30 min on ice. Bound HCAbs were detected using Alexa Fluor® 488 anti-mouse IgG antibody. Propidium iodide was used to detect the population of dead cells. Samples were analyzed using a BD LSR II flow cytometer and histograms were prepared using FlowJo software.

### Immunofluorescence (IF) analysis

Cells were grown in 4-well or 8-well chamber slides (Millipore) in their respective media. Cells were washed with PBS and fixed with 4 % paraformaldehyde (Electron Microscopy Sciences, PA, USA). Non-permeabilized cells were used to detect cell surface staining while intracellular staining was detected by permeabilizing cells with 0.1 % Triton X-100 for 15 min. 50 μg/mL of HCAbs were used to stain each cell line and bound HCAbs were detected using Alexa Fluor® 488 anti-mouse IgG antibody.

Methanol-acetone (1:1) fixed breast normal (n = 26) and tumor (n = 40) tissues were blocked with 3 % bovine serum albumin and incubated with 25-50 μg/mL of HCAb2 per section. Bound HCAb2 was detected using Alexa Fluor® 488 anti-mouse IgG antibody. Epithelial cells were detected using anti-E-cadherin antibody (1:50) and Alexa Fluor® 594 anti-rabbit IgG antibody.

MDA-MB-231 xenograft tumor sections and normal mouse tissue sections were fixed with ice-cold acetone for 20 min at -20 °C. HCAb2 localization was detected using Alexa Fluor® 488 anti-mouse IgG antibody. Anti-CD44 (1:100), anti-CD31 (1:100) and anti-calnexin (1:50) antibodies were used in respective experiments.

In all experiments nuclei were stained with 4′,6-Diamidino-2-Phenylindole, Dihydrochloride (DAPI) and images were taken using Zeiss LSM 780 confocal microscope. Images were edited using ZEN 2012 (black edition) as well as Adobe Photoshop CS4.

### Cell lysates and cell fractionation

Cells were scraped in 1 % Triton X-100/PBS or 1 % Triton X-100 + 0.1 % SDS/PBS lysis buffers supplemented with 1 mM EDTA, 0.2 mM sodium orthovanadate and fresh protease inhibitor cocktail. Nuclei were pelleted and supernatants were used for immunoprecipitation assays.

Plasma membrane protein isolation kit (SM-005, Invent Biotechnologies Inc., MN, USA) was used to fractionate cells to obtain nuclei, cytosol, organelles and plasma membrane fractions. Three P150 mm dishes with 90 % confluent cells were used to obtain cytosolic and plasma membrane fractions as per manufacturer’s instructions. Plasma membrane protein pellet was suspended in 1 % Triton X-100 + 0.1 % SDS/PBS buffer. Cytosolic fraction was brought to a final concentration of 1 % Triton X-100 and 0.1 % SDS.

Xenograft tumor pieces were placed in 500 μL of 1 % Triton X-100 + 0.1 % SDS/PBS lysis buffer supplemented with 0.2 mM sodium orthovanadate and fresh protease inhibitor cocktail (1:100) and homogenized using polytron homogenizer for 30 s - 1 min on ice. Homogenates were spun at 14,000 rpm for 20 min at 4 °C and supernatants were used for immunoprecipitation assay.

### Immunoprecipitation (IP)

Cell lysates or tumor lysates were pre-cleared with protein A beads and incubated overnight with 20 μg of HCAbs at 4 °C. Protein A beads were used to pull down heavy chain antibody-antigen complexes. Beads were boiled in sample loading buffer and proteins were resolved on a reducing SDS-PAGE gel. Proteins in the gel were stained with SYPRO® Ruby stain (Life Technologies) as per manufacturer’s instructions.

Recombinant human HSP90β (ALX-201-147-C025) was purchased from Enzo life sciences, NY, USA and resuspended in PBS. 1 μg of HSP90β in PBS along with bovine serum albumin was mixed with either 1 % Triton X-100 + 0.1 % SDS or 1 % Triton X-100 + 1 % SDS buffers and incubated overnight with 5 μg of HCAb1 and HCAb2. Immunoprecipitation was performed as explained above.

### Mass spectrometry

All analyses were performed at Keck MS and proteomics resource facility (Yale School of Medicine). In-gel trypsin digestion of proteins was performed and peptides were analyzed using LC-MS/MS on a Thermo Scientific LTQ-Orbitrap XL mass spectrometer. Mascot search algorithm was used to identify proteins from SwissProt database.

### *In vitro* scratch assay

MDA-MB-231 cells were grown to 90 % confluency in 6-well plates and serum starved for 2 h prior to forming scratches with 200 μL pipet tips. Wells were washed with PBS to get rid of floating cells and incubated with 1 mL of 1 % fetal bovine serum containing media. Cells were imaged and termed as T = 0 h images. 5 μg of HCAb1 and HCAb2 were added to respective wells and cells were imaged after 19 h (T = 19 h images).

Acellular areas at T = 0 h and T = 19 h were determined for each well using Image-Pro Plus 5.1 software. Experiment was performed in four independent wells for each treatment and area values were averaged for the 4 wells. Average acellular area at T = 0 h for each treatment was set to be 100 % and areas at T = 19 h were normalized to the corresponding average acellular area at T = 0 h. Percent acellular area remaining at T = 19 h was calculated accordingly.

### Transwell migration assay

MDA-MB-231 cells were grown to 70 % confluency and serum starved for 5 h. Cells were trypsinized and 50,000 cells were either left untreated or pre-treated with 10 μg of purified HCAb1 or HCAb2 or anti-HSP90 (sc-1055) antibody or 10 μM 17-demethoxy-17- [[2-(dimethylamino) ethyl] amino]-geldanamycin (17-DMAG, item no. 11036, Cayman chemicals, USA) for 15 min at room temperature. Cells were seeded in 200 μL of serum free DMEM with 0.1 % BSA in the upper chambers of 8 μm polycarbonate transwell inserts. Lower chambers were filled with 800 μL of serum free DMEM with 0.1 % BSA and 10 ng/mL of recombinant human EGF (PHG0311, Life technologies, USA) as a chemoattractant. Cells were allowed to migrate for 19 h at 37 °C, following which cells were fixed with 2 % paraformaldehyde for 20 min. Cells were stained with 1 % crystal violet (w/v) in 10 % ethanol for 20 min. Cells on the upper side of the membrane were removed using a cotton swab. Total numbers of cells that had migrated to the lower side of the membrane were counted. Three different inserts were used for each treatment and the average number of migrated cells was determined.

### MDA-MB-231 xenograft tumor model

MDA-MB-231 cells were trypsinized and a suspension of 1 × 10^7^ cells/mL in DMEM was prepared. Cells were pelleted and resuspended in 70 % matrigel (BD biosciences) + 30 % DMEM. 1 × 10^6^ cells were injected subcutaneously into mammary fat pad of 5 female NOD *scid* gamma (NSG) mice. After 24 days, xenograft tumors ranging from 300-500 mm^3^ were observed in all the mice. At this point, mice were retro-orbitally injected with 12 μg of purified and filtered sterile HCAb1 (n = 2) and HCAb2 (n = 3) in 100 μL of sterile saline. After 2 h, 6 h and 24 h, mice were euthanized and tumors along with various normal tissues were harvested. All animal experiments were conducted in accordance with a protocol (protocol number 100775-1016) approved by the University of Connecticut Health Center Institutional Animal Care and Use Committee.

### Small interfering RNA knockdown

siRNAs targeting luciferase (D-001100-01-20) and calnexin (ON-TARGET plus #L-003636-00-0005 SMARTpool) were purchased from GE Dharmacon, CO, USA. MDA-MB-231 cells were seeded so that they are 50-60 % confluent on the next day. siRNA lipofectamine complexes were prepared in Opti-MEM®I reduced serum media and added to cells in Opti-MEM®I reduced serum media. 6 h post transfection, cells were washed and refed with DMEM + 10 % FBS without antibiotics. After 72 h, cells were lysed in RIPA lysis buffer and 15 μg of total protein was used to perform immunoblot analysis to validate knockdown.

Based on knockdown results, mock (transfection medium alone) or 25 nM of siLuc or siCANX was transfected into MDA-MB-231 cells. After 72 h, cells were trypsinized with 0.05 % trypsin-EDTA and 0.5 × 10^6^ cells were incubated with isotype control or HCAb2. Flow cytometry was performed as explained above.

### Statistical analysis

Data from individual experiments was represented as mean ± standard deviation. Statistical analyses were performed using GraphPad Prism 5.01 and significance was determined by one-way ANOVA analysis and/or 2-tailed Student’s *t* test (* = p ≤ 0.05, ** = p ≤ 0.01).

## Results

### HCAb2 preferentially bound to the surface of MDA-MB-231 cells

**Fig. 1:**
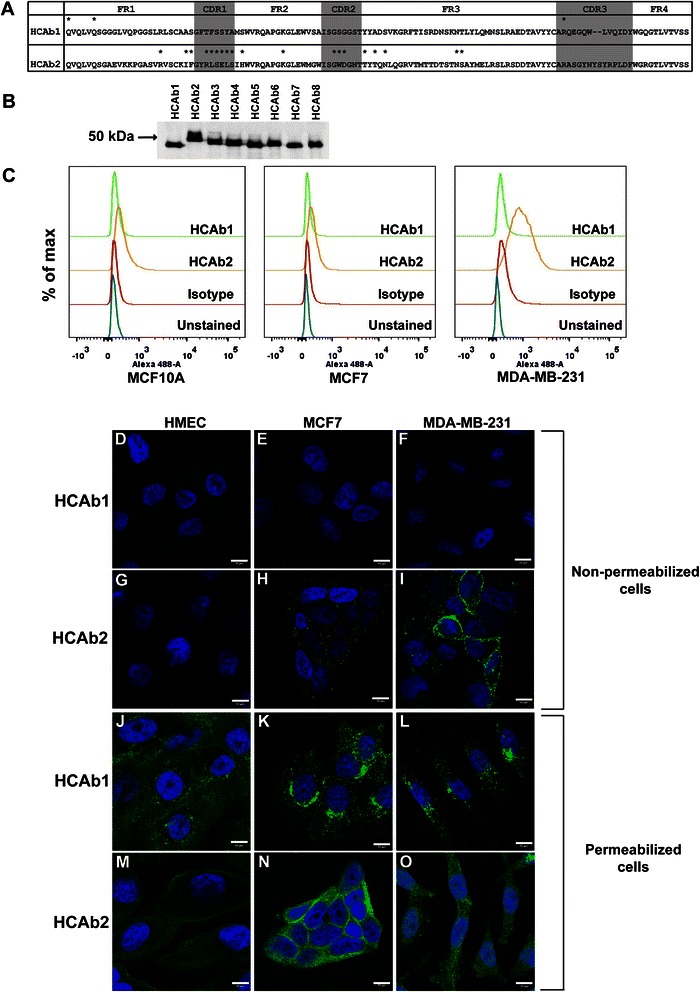
HCAb2 preferentially bound to the surface of MDA-MB-231 cells. **a**. Alignment of amino acid sequences of HCAb1 and HCAb2 revealing mutations (*) in comparison to their respective germline VDJ sequences. HCAb1 and HCAb2 nucleotide sequences were analyzed using IMGT/V-QUEST program to determine VDJ gene segments of the antibodies as well as mutations in complementarity determining regions (shaded) and framework regions. **b**. Immunoblot depicting differences in monomeric molecular weights of 8 different heavy chain antibodies. Purified heavy chain antibodies (~300 ng) were run on a reducing SDS-PAGE gel, transferred to a nitrocellulose membrane and detected using anti-mouse IgG antibody. **c**. Flow cytometry screening of MCF10A, MCF7 and MDA-MB-231 cells using HCAb1 and HCAb2. HCAb1 (green peak), HCAb2 (yellow peak), isotype control (red peak) and unstained (blue peak). **d**-**o** Immunofluorescence analysis of HMEC, MCF7 and MDA-MB-231 cells using HCAb1 and HCAb2. **d**-**i** Non-permeabilized cells were stained with HCAb1 (**d**-**f**) and HCAb2 (**g**-**i**) to determine cell surface staining. **j**-**o** Permeabilized cells were stained with HCAb1 (**j**-**l**) and HCAb2 (**m**-**o**) to determine intracellular staining. Scale bar represents 10 μm

Previously we had generated antigen-driven variable heavy chain cDNA libraries from sentinel lymph nodes of breast cancer patients [12]. Our cDNA libraries consisted of over 1100 individual variable heavy chain sequences. Using previously mentioned selection criteria [12], variable heavy chain sequences that were generated in response to antigens were determined. The selection strategy included identifying variable heavy chain sequences that were part of clonal groups and contained replacement mutations within complementarity determining regions (CDRs). Both these attributes are hallmarks of B-cells that have been activated in response to various antigens. Using this strategy, 46 different variable heavy chain sequences were identified and subcloned into a mammalian expression vector containing a C-terminal mouse Fc region. In this pilot study, 8 (out of 46) variable heavy chain sequences (HCAb1-8) derived from four breast cancer patients were selected. Variable heavy chain sequences were analyzed using IMGT/V-QUEST (http://www.imgt.org/IMGT_vquest/vquest) to determine V, D and J gene segment usage. Based on highest matching score, HCAb1 is made up of V3-23, D6-19 and J4 gene segments while HCAb2 is made up of V1-18, D5-18 and J4 gene segments. Replacement mutations in comparison to respective germline VDJ segments were determined within CDRs (1, 2 and 3) and FRs (1, 2, 3 and 4). As depicted in Fig. 1a, both HCAb1 and HCAb2 contained replacement mutations (asterisks) throughout the variable region. HCAb2 though contained a larger number of mutations selectively in CDR1 and CDR2 compared to HCAb1. We synthesized and purified bivalent HCAbs and observed that the monomeric molecular weights of HCAbs on a reducing gel approximated the expected size (50 kDa) (Fig. 1b).

Heavy chain antibodies were screened against MCF10A (non-tumorigenic cells), MCF7 (estrogen receptor positive cancer cells) and MDA-MB-231 (triple negative breast cancer cells) by flow cytometry to ensure stringent identification of cell surface targeting HCAbs. Moreover, flow cytometry allowed us to quantitatively determine the size of cell population that was targeted by HCAbs. As seen in Fig. 1c, HCAb2 (yellow peak) bound strongly to MDA-MB-231 cells (51.1 % positively stained cells) and showed weak binding to MCF10A (4.37 %) and MCF7 (0.94 %) cells compared to isotype control. HCAb1 did not bind to the surface of any of the 3 cell lines and was used as a control for subsequent experiments.

To visualize the staining pattern, immunofluorescence analysis on primary normal human mammary epithelial cells (HMEC), MCF7 and MDA-MB-231 cells was performed. HCAb1 did not show staining on the surface of HMEC (Fig. 1d), MCF7 (Fig. 1e) and MDA-MB-231 cells (Fig. 1f), while HCAb2 showed no staining on HMECs (Fig. 1g) and weak staining on MCF7 cells (Fig. 1h). HCAb2 though demonstrated definitive punctate staining on the surface of MDA-MB-231 cells (Fig. 1i). To determine if the antigen recognized by HCAb2 was also present within the cells, HCAb2 was incubated with permeabilized cells (Fig. 1m-o). HCAb2 showed reduced cytoplasmic staining in HMECs (Fig. 1m), while strong cytoplasmic staining in both MCF7 (Fig. 1n) and MDA-MB-231 cells (Fig. 1o). On the other hand, HCAb1 which did not show cell surface staining on non-permeabilized cells showed strong perinuclear staining in permeabilized HMEC (Fig. 1j), MCF7 (Fig. 1k) and MDA-MB-231 cells (Fig. 1l). Thus, both flow cytometry and immunofluorescence analysis on cells demonstrated that HCAb2 preferentially bound to the surface of MDA-MB-231 cells (Fig. 1c and i) and not to normal or MCF7 cells (Fig. 1c and g).

### HCAb2 bound strongly to primary breast tumor tissues in comparison to normal tissues

**Fig. 2:**
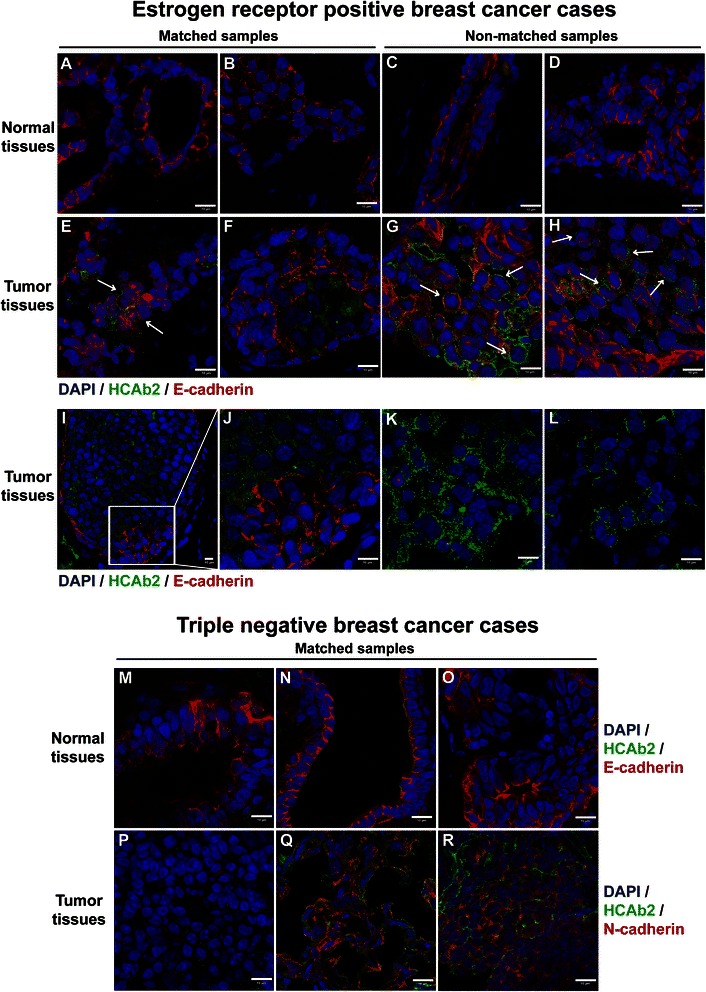
HCAb2 bound strongly to primary breast tumor tissues in comparison to normal breast tissues. **a-h** Immunofluorescence analysis of primary breast normal and ER + tumor tissues using HCAb2. Methanol-acetone (1:1) fixed normal (**a**-**d**) and ER+ tumor tissues (**e-h**) were stained with HCAb2 and E-cadherin (epithelial marker). Matched samples represent normal and tumor tissues derived from the same patient. Arrows indicate cells with positive HCAb2 staining on cell surface. Scale bar represents 10 μm. **i-l** Immunofluorescence analysis of ER+ tumor tissues using HCAb2. ER+ tumor tissues were stained with HCAb2 and E-cadherin. HCAb2 showed positive staining of E-cadherin negative tumor cells. Panel **j** is magnified view of the inset shown in panel **i**. Scale bar represents 10 μm. **m-r** Immunofluorescence analysis of matched normal and triple negative tumor tissues using HCAb2. **m-o** Normal tissues were stained with HCAb2 and E-cadherin, while tumor tissues (**p-r**) were stained with HCAb2 and N-cadherin. For all samples nuclei were stained with DAPI and the scale bar represents 10 μm

To determine if HCAb2 reveals selective binding to human breast tumors, a cohort of fresh frozen human breast tissue samples was screened with HCAb2. Our cohort consisted of 31 estrogen receptor alpha positive (ER+) breast tumor cases, of which 12 had matching normal/non-tumor tissues from the same surgical resection. The cohort also consisted of 5 triple negative tumor cases with 4 matched normal/non-tumor tissues and 4 human epidermal growth factor receptor 2 positive (HER2+) tumor cases with 3 matched normal/non-tumor tissues. Immunofluorescence analysis revealed that HCAb2 showed no or weak staining on all of the 26 normal breast tissues (Fig. 2a-d and m-o). Examination of ER+ tumor tissues revealed heterogenous staining pattern ranging from a few positively stained cells to those with large clusters of positively stained cells (Fig. 2e-h). HCAb2 staining on ER+ tumor samples was found to be selective to epithelial cells as evidenced by positive E-cadherin staining (Fig. 2e, g and h). In addition, we observed that HCAb2 showed cell surface staining on tumor tissues (Fig. 2g-h). Interestingly on some of the ER+ tumor samples, HCAb2 showed strong staining on E-cadherin negative epithelial cells (Fig. 2i-l). This can be clearly observed in Fig. 2i as well as Fig. 2j (magnified view), wherein HCAb2 strongly stained E-cadherin negative cells while weakly stained E-cadherin positive cells within the same cluster. Overall we observed that 12 of the 31 ER+ tumor samples, 3 of the 5 triple negative tumor samples and 1 of the 4 HER2+ tumor samples showed positive staining with HCAb2. For each of the 3 basic tumor types, approximately 5-10 % of tumor epithelial cell populations were found to be positively stained by HCAb2, thus indicating that HCAb2 bound specifically to only a subset of tumor cells in each tumor. Also in a majority of tumor samples, HCAb2 staining was observed to be punctate in nature (Fig. 2g-h and k-l), similar to the pattern observed with MDA-MB-231 cells (Fig. 1i).

In triple negative tumor tissues, HCAb2 showed moderate (Fig. 2p) to strong staining (Fig. 2q-r) on N-cadherin positive tumor cells. Similar to ER+ tumor samples, HCAb2 showed preferential staining on triple negative tumor tissues (Fig. 2p-r) in comparison to patient matched normal tissues (Fig. 2m-o). HER2+ tumor tissues did not show strong staining with HCAb2 and only 1 of the 4 samples revealed moderate staining with HCAb2. Immunofluorescence analyses on primary breast tissues demonstrated that HCAb2 preferentially bound to tumor tissues in comparison to normal/non-tumor tissues and thereby enabled HCAb2 as a lead antibody for further characterization.

### Identification of target antigens of HCAb1 and HCAb2

**Fig. 3:**
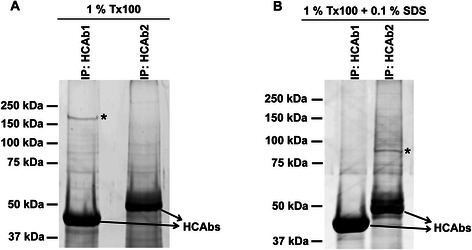
Identification of target antigens of HCAb1 and HCAb2. **a** and **b** Immunoprecipitation of respective target antigens by HCAb1 and HCAb2. 20 μg of HCAb1 and HCAb2 were used to immunoprecipitate the target antigens from 1 % Triton X-100 (**a**) or 1 % Triton X-100 + 0.1 % SDS (**b**) MDA-MB-231 lysates. Immunoprecipitated proteins were run on a reducing SDS-PAGE gel and proteins in the gel were stained with SYPRO® Ruby stain. * indicates specific band for HCAb1 (**a**) and HCAb2 (**b**)

In order to determine the antigens recognized by HCAb1 and HCAb2, immunoprecipitation of antigens followed by protein identification using mass spectrometry was performed. We chose MDA-MB-231 cell lysates since the respective target antigens for both HCAb1 (Fig. 1l) and HCAb2 (Fig. 1i) were abundant in MDA-MB-231 cells. Immunoprecipitated proteins were run on a reducing SDS-PAGE gel and visualized using SYPRO® Ruby stain. Immunoprecipitation with HCAb1 revealed a specific band (~MW 200 kDa) from 1 % Triton X-100 lysates, while immunoprecipitation with HCAb2 did not show any specific band under these conditions (Fig. 3a). On the other hand, immunoprecipitation with HCAb2 revealed a specific band (~MW 90 kDa) from 1 % Triton X-100 lysates supplemented with 0.1 % SDS (Fig. 3b). Furthermore, these buffer conditions were unfavorable for HCAb1 to immunoprecipitate the band seen previously in buffer lacking SDS (Fig. 3a). Multiple repeats revealed similar results and led us to conclude that the interaction between HCAb1 and its antigen is abolished in the presence of 0.1 % SDS, while the interaction between HCAb2 and its antigen requires the presence of 0.1 % SDS. This could suggest that the antigen recognized by HCAb2 requires SDS to be solubilized in cell lysates.

Specific gel bands observed in Fig. 3a and b were excised and the tryptic peptides were analyzed by mass spectrometry. Peptides identified from this analysis were used to search SwissProt database to obtain a list of potential target proteins. Tables 1 and 2 show the top five proteins identified from HCAb1 and HCAb2 immunoprecipitated bands, respectively. Keratin proteins observed in the analysis are typical contaminants and were probably introduced during handling of gel bands. The top hit for HCAb1 was clathrin heavy chain 1 (CLTC) protein with the identified peptides covering 40.7 % of the protein (Table 1). Molecular weight of CLTC protein (191.493 kDa) is similar to molecular weight of the excised gel band (Fig. 3a). This would suggest that the target antigen of HCAb1 could be CLTC protein. Similarly, the top hits for HCAb2 were heat shock protein HSP90-beta and heat shock protein HSP90-alpha with peptide coverage of 51.7 and 40.6 %, respectively (Table 2). Molecular weight of the excised band (~90 kDa) (Fig. 3b) overlaps with molecular weights of both the HSP90 isoforms (Table 2), suggesting that the target antigen of HCAb2 could be HSP90.

**Table 1:** List of top five proteins identified from HCAb1 specific gel band

Score	Gene name	Swiss-prot accession no.	Protein name	MW (Da)	% coverage
2366	CLTC	Q00610	Clathrin heavy chain 1	191493	40.7
776	KRT1	P04264	Keratin 1	66027	24.4
720	-	-	Unnamed protein product	59492	27.3
682	KRT1	P04264	Keratin 1	66026	24.4
463	KRT2	P35908	Epidermal cytokeratin 2	65825	27.8

**Table 2:** List of top five proteins identified from HCAb2 specific gel band

Score	Gene name	Swiss-prot accession no.	Protein name	MW (Da)	% coverage
1812	HSP90AB1	P08238	Heat shock protein HSP 90-beta	83212	51.7
1007	KRT16	P08779	Keratin, type I cytoskeletal 16	51236	45
1004	HSP90AA1	P07900	Heat shock protein HSP 90-alpha	84607	40.6
980	KRT6C	P48668	Keratin, type II cytoskeletal 6C	59988	30.7
970	KRT6A	P02538	Keratin, type II cytoskeletal 6A	60008	30.7

### Validation of target antigens of HCAb1 and HCAb2

Clathrin heavy chain was found to be the putative target antigen of HCAb1 (Table 1) and to validate this, co-localization immunofluorescence analysis was performed on permeabilized MCF7 cells using HCAb1 and a commercial anti-CLTC antibody. As seen in Additional file 1: Figure S1, HCAb1 and the commercial antibody co-localized to the peri-nuclear region, suggesting that HCAb1 binds to clathrin heavy chain.

**Fig. 4:**
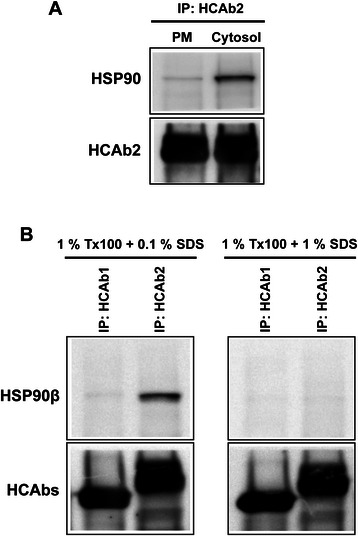
Validation of target antigens of HCAb1 and HCAb2. **a** Validation of HSP90 to be the target antigen of HCAb2. Immunoprecipitation of HSP90 was performed from cytosolic and plasma membrane (PM) fractions using HCAb2. Immunoprecipitated HSP90 was detected using a commercial anti-total HSP90 antibody. **b** Immunoprecipitation of recombinant human HSP90β protein using HCAb1 and HCAb2. HCAb1 and HCAb2 (5 μg each) were used to immunoprecipitate recombinant HSP90β (1 μg) resuspended in either 1 % Triton X-100 + 0.1 % SDS or 1 % Triton X-100 + 1 % SDS buffers. Immunoprecipitated HSP90β protein was detected using a commercial anti-total HSP90 antibody. Equal amounts of HCAb1 and HCAb2 were pulled down as detected by anti-mouse IgG antibody

From mass spectrometric analysis (Table 2), HSP90 was found to be the antigen for HCAb2. HSP90 is an intracellular molecular chaperone that aids in appropriate folding of a wide variety of proteins [19]. Four different isoforms of HSP90 are present which include HSP90α and HSP90β (cytosolic isoforms), Grp94 (endoplasmic reticulum isoform) and TRAP1 (mitochondrial isoform) [20]. In addition to its cytosolic localization, HSP90 has been shown to be present on plasma membrane of cells as well as in the extracellular space [21, 22]. Indeed HSP90 has been shown previously to be on the surface of MDA-MB-231 cells [23]. In order to validate HSP90 to be the target antigen of HCAb2, MDA-MB-231 cells were fractionated to obtain plasma membrane and cytosolic fractions that were subsequently used for immunoprecipitation with HCAb2. Immunoprecipitated proteins were run on a reducing SDS-PAGE gel and immunoblot analysis was performed using a commercial anti-HSP90 antibody. Commercial anti-HSP90 antibody used for immunoblot analysis detected the levels of total HSP90 as it binds to both the isoforms (HSP90α and HSP90β). As seen in Fig. 4a, HCAb2 pulled down HSP90 from both fractions with higher amounts being pulled down from the cytosolic fraction. Differences in the amount of HSP90 being pulled down could be attributed to the abundance of HSP90 in cytosol compared to plasma membrane. Equal amounts of HCAb2 were pulled down in both immunoprecipitations as detected with anti-mouse antibody (Fig. 4a).

The list of potential target proteins identified by HCAb2 contained both HSP90α and HSP90β isoforms with HSP90β being the highest scoring protein (Table 2). This suggests that HCAb2 can bind to both isoforms or that the peptides identified from mass spectrometric analysis were common to both isoforms (amino acid sequence homology - 85.8 %). Analysis of mass spectrometric results revealed a total of 43 peptides that matched to HSP90 protein. Of the 43 peptides, 14 peptides were unique to HSP90α and 20 peptides were unique to HSP90β while the remaining 9 peptides were common to both isoforms (Additional file 2: Table S1). Due to identification of a higher percentage of unique peptides, HSP90β could be the target antigen of HCAb2. To determine specificity of HCAb2, immunoprecipitation of recombinant human HSP90β was performed with HCAb2 and HCAb1 (control). Immunoprecipitated HSP90β was detected using a commercial anti-HSP90 antibody. As seen in Fig. 4b, HCAb2 pulled down HSP90β strongly in the presence of 1 % Triton X-100 and 0.1 % SDS. This interaction was abolished when SDS concentration was elevated from 0.1 % to 1 %. A faint diffuse band was seen from immunoprecipitation with HCAb1 (1 % Tx100 + 0.1 % SDS) as well as for both HCAb1 and HCAb2 (1 % Tx100 + 1 % SDS), which could be due to a minor non-specific interaction. Taken together these results validate that the target antigen of HCAb2 is HSP90, that HCAb2 is capable of recognizing both cytosolic and plasma membrane associated HSP90 protein and that a small amount of SDS was required to facilitate the interaction between HCAb2 and HSP90.

### HCAb2 affected *in vitro* migration of MDA-MB-231 cells

**Fig. 5:**
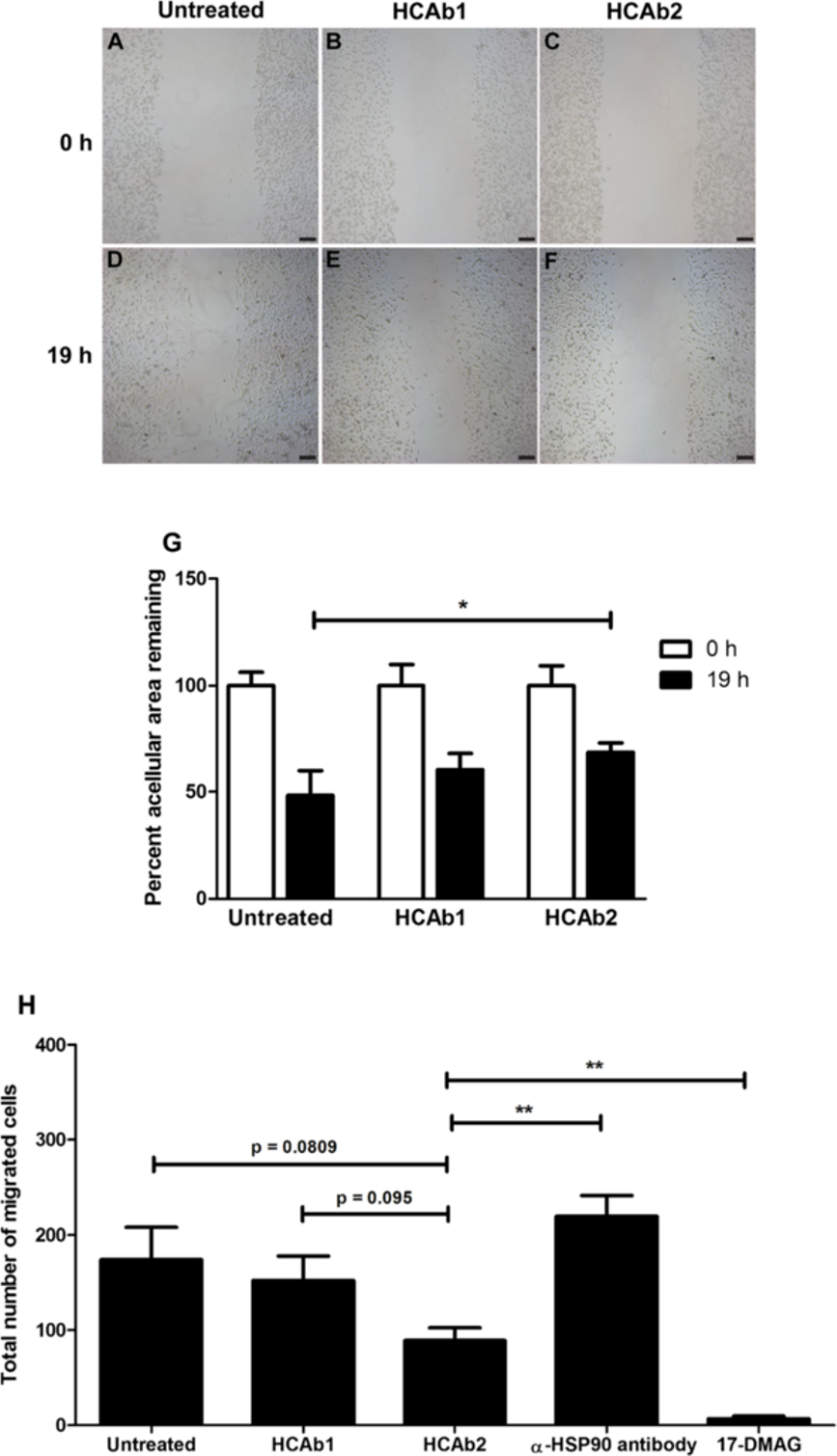
HCAb2 reduced *in vitro* migration of MDA-MB-231 cells. **a-f** Representative images of scratch assay, T = 0 h (panels **a-c**) and T = 19 h (panels **d-f**). Scratches were made using 200 μL pipet tips and T = 0 h images were taken. Subsequently cells were left untreated or incubated with HCAb1 and HCAb2 (5 μg each) and imaged after 19 h. Scale bar represents 100 μm. **g** Quantification of percent acellular area remaining after 19 h of treatment with HCAb1 and HCAb2. Acellular area at T = 0 h and T = 19 h was determined for each well using Image-Pro software (n = 4 wells per treatment). Average area at T = 0 h for each treatment was set to be 100 % and areas at T = 19 h were normalized to the corresponding average acellular area at T = 0 h. Percent acellular area remaining was calculated accordingly. Error bars represent standard deviation and statistical significance was determined by Student’s *t* test, * = p ≤ 0.05. **h** Quantification of cell migration in a transwell assay. MDA-MB-231 cells were left untreated or pre-treated with 10 μg of HCAb1 or HCAb2 or anti-HSP90 antibody (sc-1055) or 10 μM 17-DMAG for 15 min at room temperature. Cells were allowed to migrate for 19 h at 37 °C with EGF as the chemoattractant. Cells were stained with crystal violet and total numbers of migrated cells were counted. Average number of migrated cells with standard deviation was plotted for 3 transwell inserts per treatment. Statistical significance was determined using Student’s *t* test, ** = p ≤ 0.01

Cell surface and extracellular localized HSP90 has been implicated in increased invasiveness of tumors [24–30]. Levels of secreted HSP90α have been shown to be positively correlated with malignancy of different tumor types [24]. Cell impermeable anti-HSP90 antibody and a cell impermeable small molecule inhibitor of HSP90 have both been shown to reduce tumor cell motility and invasion [31–33]. Since HCAb2 binds to cell surface HSP90, we aimed to determine if HCAb2 could reduce migration of MDA-MB-231 cells. An *in vitro* scratch assay was performed with MDA-MB-231 cells in the presence of HCAb1 and HCAb2 and compared to untreated controls. Representative images were taken at T = 0 h and T = 19 h to determine potential differences in migration distances with different treatments. All the wells had similar scratch areas at T = 0 h (Fig. 5a-c) while at T = 19 h, HCAb2 treated wells (Fig. 5f) showed reduced migration of cells into acellular area in comparison to untreated (Fig. 5d) or HCAb1 (Fig. 5e) treated wells. In order to quantify differences in migration, acellular area at 0 h and at 19 h was determined. Percent acellular area remaining after 19 h was determined and values from 4 different wells were averaged. As seen in Fig. 5g, percent acellular area remaining after 19 h was highest in HCAb2 treated wells (68.46 %) and lowest in untreated wells (48.29 %), while HCAb1 treated wells had 60.3 % of acellular area still remaining. This reduction could be due to internalization of HCAb1 leading to an indirect effect on migration [34]. Percent acellular area remaining at 19 h between untreated and HCAb1 treated cells was not significantly different (p = 0.1336) nor was the difference between HCAb1 and HCAb2 treated cells (p = 0.1124). Percent acellular area remaining between untreated and HCAb2 treated cells was significantly different (p = 0.0173), suggesting that HCAb2 was able to reduce migration of MDA-MB-231 cells.

To further test the efficiency of HCAb2 in inhibiting migration of MDA-MB-231 cells, an *in vitro* transwell migration assay was performed. MDA-MB-231 cells were serum starved for 5 h, following which cells were left untreated or pre-treated with either 10 μg of HCAb1 or HCAb2. Our previous analysis with three different commercial anti-HSP90 antibodies (CST #4877, sc-1055 and sc-1057) revealed that these antibodies did not bind to cell surface HSP90 but bound to intra-cellular HSP90 (Additional file 3: Figure S2). Therefore, one of the commercial antibodies (sc-1055) was used as a negative control in the transwell migration assay. Also a positive control was required to determine the extent of inhibition by HCAb2. To this end, 17-DMAG (HSP90 inhibitor), which has been shown to inhibit migration [33, 35, 36] was used at 10 μM to determine the maximal inhibition of migration. As shown in Fig. 5h, HCAb2 reduced migration of MDA-MB-231 cells in comparison to untreated and HCAb1 treated cells. Relative to untreated cells, HCAb2 revealed 48.85 % inhibition of migration while HCAb1 only showed 12.64 % inhibition of migration. Unfortunately, results with HCAb2 did not reach statistical significance in comparison to untreated (p = 0.0809) and HCAb1 (p = 0.095) treated cells but showed trend towards significance. HCAb2 though significantly reduced migration of MDA-MB-231 cells in comparison to the commercial anti-HSP90 antibody (p = 0.007). Similar levels of significance were not evident with either untreated (p = 0.3233) or HCAb1 treated cells (p = 0.116) in comparison to the commercial anti-HSP90 antibody treated cells. Although HCAb2 reduced migration of MDA-MB-231 cells, HCAb2 was not as effective as the HSP90 inhibitor (17-DMAG), which revealed 96.16 % inhibition of migration. This could be due to the fact that HCAb2 binds to cell membrane associated HSP90, while 17-DMAG could be targeting total HSP90. Therefore, binding of HCAb2 to HSP90 could inhibit invasiveness of MDA-MB-231 cells at a moderate yet significant level. The potential of HCAb2 in inhibiting *in vivo* tumor metastasis will be performed as a part of future directions.

### HCAb2 preferentially localized to MDA-MB-231 xenograft tumors

**Fig. 6:**
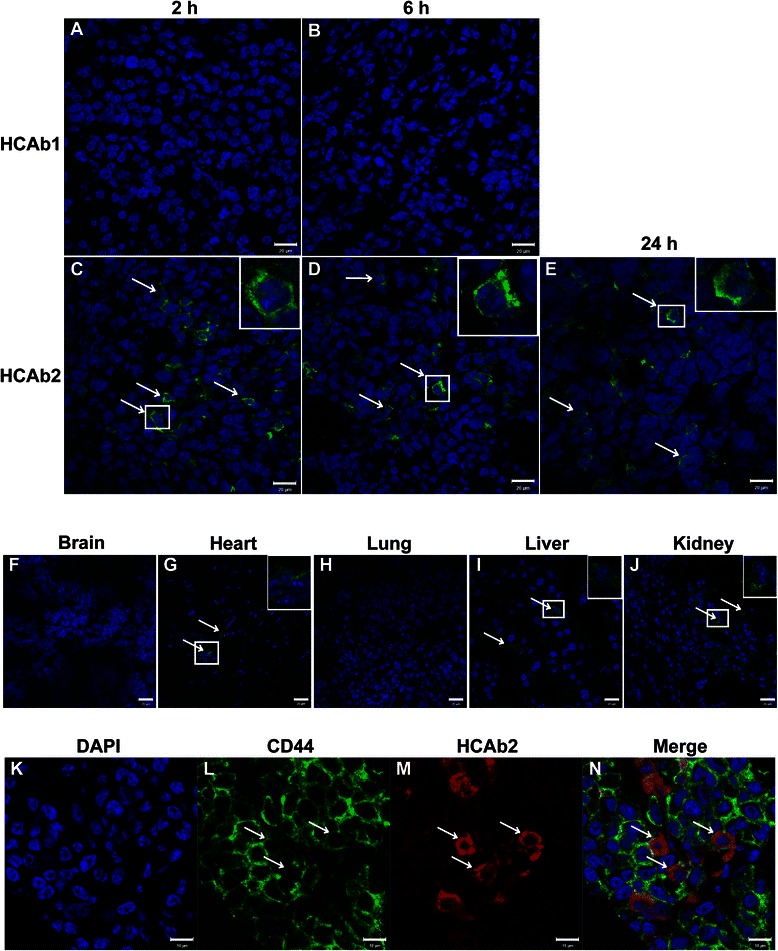
HCAb2 localized specifically to MDA-MB-231 xenograft tumors in immunodeficient mice. **a-e** Representative images showing localization of HCAb2 to tumors at 2 h, 6 h and 24 h time points. Female NSG mice bearing tumors ranging from 300-500 mm^3^ were retro-orbitally injected with 12 μg of HCAb1 (n = 2) and HCAb2 (n = 3) into respective animals. After 2 h, 6 h and 24 h mice were euthanized and tumors along with various normal tissues were stained to detect HCAb1 and HCAb2 localization. HCAb1 did not localize to the tumors (panels **a** and **b**), while HCAb2 localized to tumors at the earliest time point (panel **c**) and the later time points of 6 h (panel **d**) and 24 h (panel **e**). Arrows indicate cells with HCAb2 staining. Insets reveal magnified image and scale bar represents 20 μm. **f-j** Immunofluorescence analysis of mouse normal tissues to determine distribution of HCAb2. Frozen sections of brain (panel **f**), heart (panel **g**), lung (panel **h**), liver (panel **i**) and kidney (panel **j**) tissues from 24 h time point mouse were analyzed to detect the presence of HCAb2. Low levels of HCAb2 were detected in heart (panel **g**), liver (panel **i**) and kidney tissues (panel **j**). Arrows indicate HCAb2 localization. Insets reveal magnified image and scale bar represents 20 μm. **k**-**n** Immunofluorescence analysis of xenograft tumor tissue to identify HCAb2 localization in MDA-MB-231 cells. 24 h time point tumor section was incubated with anti-CD44-FITC and Alexa Fluor® 594 anti-mouse IgG antibodies. From panels **l**, **m** and **n**, it can be observed that HCAb2 localizes to MDA-MB-231 cells. Arrows indicate cells with positive CD44 and HCAb2 staining. Scale bar represents 10 μm

Leading up to this point, we observed that HCAb2 is a tumor specific antibody (Figs. 1 and 2) that binds to cell surface HSP90 (Fig. 4a) and inhibits invasiveness of tumor cells *in vitro* (Fig. 5). We then wanted to determine if HCAb2 can target tumors in an *in vivo* xenograft model. To this end, MDA-MB-231 xenograft tumors ranging from 300-500 mm^3^ were generated in female NSG mice. Following which, 12 μg of HCAb1 and HCAb2 were injected retro-orbitally into circulation of tumor-bearing animals. After 2 h, 6 h and 24 h post-injection of HCAb1 (control) and HCAb2 into respective animals, xenograft tumors and normal tissues were harvested and screened to determine localization of HCAb1 and HCAb2. As expected, HCAb1 did not localize to xenograft tumors at either of the time points (2 h and 6 h) (Fig. 6a-b), while HCAb2 was found in tumors at 2 h (Fig. 6c), 6 h (Fig. 6d) and 24 h (Fig. 6e) time points. In different xenograft tumors, only small populations of cells were observed to stain positively for HCAb2 while vast majority of the tumors were negative for HCAb2 localization.

To determine the distribution of HCAb2 in non-tumor mouse tissues, brain, heart, lung, liver and kidney tissues at the 24 h time point were examined. As seen in Fig. 6f and h, HCAb2 was not detected in brain or lung tissues, respectively while heart (Fig. 6g), liver (Fig. 6i) and kidney tissues (Fig. 6j) revealed weak staining for HCAb2. Detection of HCAb2 in kidney tissue could be due to renal clearing of injected HCAb2. These results indicate that HCAb2 preferentially localized to xenograft tumors in comparison to normal tissues.

To confirm that HCAb2 specifically targeted MDA-MB-231 cells within xenograft tumors and not mouse cells, a 24 h time point tumor section was probed with anti-CD44-FITC and Alexa Fluor® 594 anti-mouse IgG antibodies (note: there is little circulating IgG in NSG mice). MDA-MB-231 cells are CD44+ and we observed that the majority of xenograft tumor cells were CD44+ MDA-MB-231 cells (Fig. 6l) with very few mouse cells. Also HCAb2 localized cells were positive for CD44 staining (Fig. 6l-n) but had lower levels of CD44 compared to cells without HCAb2 localization. This would confirm that HCAb2 specifically localized to MDA-MB-231 xenograft tumor cells. HCAb2 was also observed to be present in cytosol of targeted cells (Fig. 6n), suggesting that the bound HCAb2 was internalized by target cells.

We also wanted to determine if HCAb2 can bind to HSP90 from xenograft tumor lysates. Xenograft tumor pieces were homogenized and incubated with exogenous HCAb1 (control) and HCAb2. Immunoprecipitated proteins were detected using a commercial anti-HSP90 antibody. It was readily apparent that HCAb2 pulled down HSP90 from tumor lysates, while HCAb1 did not pull down HSP90 at all (Additional file 4: Figure S3). In summary, we observed that HCAb2 bound preferentially to a small population of MDA-MB-231 xenograft tumor cells in comparison to mouse normal tissues and also bound to HSP90 from xenograft tumor lysates.

### HCAb2 localized to a subset of MDA-MB-231 xenograft tumor cells

**Fig. 7:**
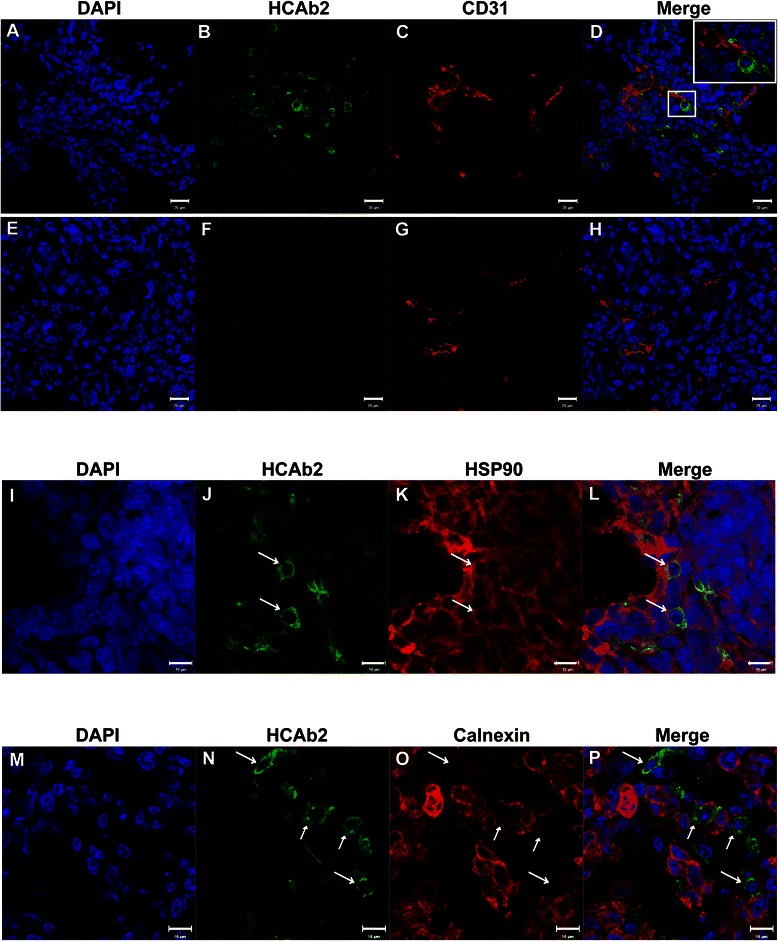
HCAb2 specifically targeted a unique population of MDA-MB-231 xenograft tumor cells. **a-h** Immunofluorescence analysis of xenograft tumor vasculature and HCAb2 localization. 24 h time point tumor section was incubated with anti-CD31 and Alexa Fluor® 488 anti-mouse IgG antibodies. Nuclei were stained with DAPI. (**a-d)** Representative images depicting a region in the tumor that shows HCAb2 localization and positive CD31 staining. (**e-h)** Representative images depicting a different region in the same tumor showing no HCAb2 localization but positive CD31 staining. Scale bar represents 20 μm. **i-l** Immunofluorescence detection of HSP90 and HCAb2 in xenograft tumor cells. 24 h time point tumor section was incubated with anti-HSP90 and Alexa Fluor® 488 anti-mouse IgG antibodies. Isolated HCAb2 localization was observed (panel **j**) while uniform HSP90 staining was observed (panel **k**) throughout the tumor section. Scale bar represents 10 μm. **m-p** Immunofluorescence detection of calnexin and HCAb2 in xenograft tumor cells. 24 h time point tumor section was incubated with anti-calnexin and Alexa Fluor® 488 anti-mouse IgG antibodies. HCAb2 localized to MDA-MB-231 cells that lacked calnexin staining as seen from panels **n-p**. Scale bar represents 10 μm

As seen from Fig. 6c-e and n, HCAb2 localized to a small population of MDA-MB-231 cells within the tumors. To determine if differential vascularization of tumors could be responsible for unique localization of HCAb2; CD31 and HCAb2 staining was performed on a 24 h tumor section (Fig. 7a-h). We observed that HCAb2 localized to cells that were in close proximity to blood vessels (Fig. 7b-d) suggesting that these cells were accessible to HCAb2. But even within this section, a majority of the cells that were in close proximity to blood vessels were not found to be positive for HCAb2 localization. This effect was seen more prominently in other fields of the same tumor (Fig. 7e-h) wherein vascularized regions of the tumor (Fig. 7g and h) did not show any HCAb2 localization (Fig. 7f). This result eliminates possibility that the unique localization of HCAb2 was solely due to reduced accessibility to tumor cells in specific areas.

To determine if unique localization of HCAb2 was due to differential expression of HSP90 in tumor cells, a 24 h time point tumor section was probed with a commercial anti-HSP90 and Alexa Fluor® 488 anti-mouse IgG antibodies. HCAb2-localized tumor cells did not show any changes in the intracellular levels of HSP90 (Fig. 7i-l), however the levels of cell surface HSP90 could have been increased. To understand the factors that could lead to increased cell surface expression of HSP90, we focused on the function of HSP90 in tumor cells. HSP90 along with ER chaperones calnexin, calreticulin and protein disulfide isomerase aids in the folding of various proteins. Variation in the levels of different chaperones could lead to generation of misfolded proteins and in turn lead to stress at the level of protein folding. Previously, calnexin deficient cells have been shown to be in a state of constant stress and have constitutively active unfolded protein response [37]. Furthermore, cells that are undergoing stress have been shown to translocate HSP70 from cytosol to plasma membrane [38]. This suggests that the cell surface expression of HSP90, similar to HSP70 could be increased in cells that are undergoing stress. Indeed a previous study has shown that the levels of cell surface HSP90 were increased in a glioblastoma multiforme cell line subjected to hypoxic conditions [39]. We therefore hypothesized that the xenograft tumor cells with HCAb2 localization were highly stressed and thereby had increased levels of HSP90 on cell surface. To test this hypothesis, a 24 h time point MDA-MB-231 tumor section was stained to visualize HCAb2 and calnexin. Cells that were positive for HCAb2 were observed to be negative for calnexin staining (Fig. 7m-p). Of note, another chaperone marker protein disulfide isomerase (PDI) was unchanged in HCAb2 positive tumor cells (Additional file 5: Figure S4). This surprising result suggested that HCAb2 specifically targeted a unique population of cells that have significant stress with respect to metabolic or unfolded protein response events. It is unclear as to the significance or the cause of reduced levels of calnexin in these tumor cells. Loss of calnexin could also explain the reduced levels of CD44 on cells that showed HCAb2 localization (Fig. 6k-n). Immunofluorescence analysis on MDA-MB-231 cells in culture did not reveal any cells that showed reduced levels of calnexin or protein disulfide isomerase (Additional file 6: Figure S5). This suggests that the loss of calnexin in xenograft tumor cells occurred during tumor formation and/or within the tumor microenvironment.

Finally, we wanted to determine if reduction in levels of calnexin could lead to increased cell surface expression of HSP90. To this end, calnexin was knocked down in MDA-MB-231 cells (Additional file 7: Figure S6A) and HCAb2 binding to cells was determined. Confirmed calnexin knock down led to a moderate increase in cell surface binding of HCAb2 (Additional file 7: Figure S6B). This suggests that reduction in levels of calnexin alone is not sufficient to affect cell surface expression of HSP90 in cell culture environment.

## Discussion

The goal of this study was to identify cell surface targeting tumor specific antibodies derived from responsive patient sentinel lymph nodes. To attain this goal, 8 heavy chain antibodies were screened from a pool of 46 selected antigen-driven antibodies. Owing to the fact that HCAb1 did not bind to a cell surface antigen, nor did it show differential staining between primary human breast normal and tumor tissues (data not shown), HCAb1 was ruled out as a tumor specific antibody. However, since HCAb1 did not show cell surface staining, it was used as a negative control throughout the study. Using stringent screening procedures, HCAb2 was identified as a cell surface HSP90 targeting heavy chain antibody. HSP90 is an abundant intracellular chaperone (2-3 % of total protein) whose expression increases in stressed cells [40, 41]. Such high levels of HSP90 are postulated to be necessary for efficient folding of a multitude of proteins. HSP90 has been shown to be upregulated in a wide variety of tumors including breast tumors [42] to aid in folding and stabilization of various over-expressed or mutant tumor associated proteins such as EGFR [43], mutant B-Raf [44], mutant BRCA1 [45] and mutant p53 [46]. High expression of HSP90 is therefore an essential requirement for survival of tumor cells and has been shown to correlate with reduced survival in breast cancer patients [42]. Indeed HSP90 inhibitors have been shown to downregulate the expression of mutant epidermal growth factor receptor in tumors [47] and selectively kill tumor cells [48]. HSP90 inhibitors such as DMAG and 17-AAG [33, 49–51] have shown promising results, with 17-AAG showing anti-cancer activity in a phase II trial [52].

In addition to intracellular localization of HSP90, numerous reports have indicated the presence of HSP90 on cell surface as well as in extracellular space of tumors [21, 22]. HCAb2 showed cytosolic staining in HMEC, MCF7 and MDA-MB-231 cells but showed cell surface staining only on aggressive MDA-MB-231 cells. Indeed HSP90 has been previously shown to be present on the surface of MDA-MB-231 cells [53]. Membrane associated HSP90 can activate HER-2 and also interact with Cdc37 leading to increased invasiveness of cancer cells [27, 53]. Extracellular HSP90 has also been shown to activate matrix metalloproteinase-2 [28] and plasminogen [54] leading to increased cell motility. The actual mechanism by which HSP90 gets to cell surface or is released outside the cells is still unclear, with some evidence pointing to the role of exosomes [54]. MDA-MB-231 cells undergoing hypoxic stress have been shown to release increased levels of exosomes [55]. This surge in exosomal release during stress conditions could result in higher amounts of extracellular HSP90 and in turn lead to increased invasiveness of cells. It is interesting to note that HCAb2 demonstrates punctate staining pattern on MDA-MB-231 cells as well as primary human breast tumor tissues. The punctate pattern very well could represent vesicular structures/exosomes since HSP90 has been shown to be present in exosomes derived from bladder and colorectal cancer cells [56, 57].

Considering the significance of membrane bound HSP90 in tumor metastasis, it is possible that targeting cell surface HSP90 may suppress tumor metastasis. Indeed DMAG-N-oxide, a cell impermeable HSP90 inhibitor has been shown to inhibit migration of B16 melanoma cells as well as their lung colonization [33]. Along similar lines, we have shown that HCAb2 was also able to reduce migration of MDA-MB-231 cells *in vitro*. The advantages of using an antibody such as HCAb2 to target cell surface HSP90 over a small molecule inhibitor would be activation of immune effector functions such as antibody-dependent cell-mediated cytotoxicity and complement-dependent cytotoxicity. The ability of HCAb2 in performing these effector functions will be tested in our future experiments.

Presence of cell surface HSP90 seems to aid in increasing the invasiveness of tumor cells. But the reasons for cell surface localization are not clearly understood. HSP70 is also an intracellular chaperone that is overexpressed in tumors and localizes to plasma membrane of stressed cells [38, 58]. Similar to HSP70, HSP90 may also be translocated to plasma membrane of stressed tumor cells. From our screening analysis on primary human breast tumor tissues, we observed that HCAb2 revealed staining of isolated cells or clusters of cells within the tumors. It could be possible that these isolated or clusters of cells were exposed to different microenvironmental stresses leading to increased cell surface expression of HSP90. This feature of HCAb2 binding to stressed cells was also observed in MDA-MB-231 xenograft tumors. HCAb2 localization in xenograft tumors was restricted to a small subset of cells that were deficient for calnexin. Calnexin along with other ER chaperones including calreticulin and protein disulfide isomerase maintains protein homeostasis and any perturbations to this system could lead to cellular stress. Cellular stresses in tumors can lead to generation of misfolded proteins, which if left unresolved can activate unfolded protein response. Previous studies have shown that cells with reduced calnexin have constitutively active unfolded protein response [37]. This strengthens our argument that HCAb2 localized to highly stressed cells with increased cell surface HSP90.

Calnexin aids in folding of MHC class I molecules and may also aid in loading of peptides onto MHC class I molecules [59]. Interestingly, calnexin has been shown to be downregulated in brain metastases of breast tumors compared to unpaired primary breast lesions [60] as well as in metastatic melanoma lesions in comparison to primary melanoma lesions [61]. Down regulation of calnexin can lead to reduced MHC class I molecules on cell surface and has been hypothesized to aid cells in escaping from adaptive immune response. We believe that HCAb2 is an ideal antibody to target metastatic tumor cells since HCAb2 binds to cell surface HSP90 as well as to cells that are deficient in calnexin. The relationship between reduced calnexin and increased HSP90 on cell surface needs to be further evaluated. Preliminary results with calnexin knockdown (Additional file 6: Figure S5) indicated that this relationship is not direct or causal and is probably accentuated in an *in vivo* tumor with heterogenous microenvironmental stresses.

## Conclusions

In conclusion, we have developed a powerful strategy whereby a library of patient-derived heavy chain antibodies can be screened to identify tumor targeting antibodies. Identification of HCAb2 validates the strength of this research strategy. The antigen for HCAb2 was found to be HSP90 and HCAb2 bound to a unique subset of xenograft tumor cells that were negative for calnexin. This raises interesting questions regarding the connection between reduced levels of calnexin and increased expression of cell surface HSP90. In addition, HCAb2 can be a unique reagent to target aggressive human tumor cells *in vivo* and may be useful for therapeutic applications.

## Additional files

Additional file 1: Figure S1. Clathrin heavy chain protein is the target antigen of HCAb1. A-D. MCF7 cells were incubated with HCAb1 and a commercial anti-clathrin heavy chain antibody. Arrows indicate regions that show co-localization of HCAb1 and anti-clathrin heavy chain antibody. Nuclei were stained with DAPI. Scale bar represents 10 μm. (TIFF 2942 kb)
Additional file 2: Table S1. List of different HSP90 peptides identified from HCAb2 immunoprecipitated gel band using mass spectrometry. (XLSX 14 kb)
Additional file 3: Figure S2. Commercial anti-HSP90 antibodies did not bind to cell surface HSP90. Immunofluorescence analysis of MDA-MB-231 cells was performed with 3 different commercial anti-HSP90 antibodies. Commercial anti-HSP90 Ab 1 (sc-1055), commercial anti-HSP90 Ab 2 (sc-1057) and commercial anti-HSP90 Ab 3 (CST 4877) were used as indicated. Non-permeabilized cells were used to determine cell surface staining and permeabilized cells were used to determine intracellular staining. Nuclei were stained with DAPI. Scale bar represents 10 μm. (TIFF 2948 kb)
Additional file 4: Figure S3. Immunoprecipitation of HSP90 from xenograft tumor lysates using HCAb2. RIPA lysates of xenograft tumor pieces were used for immunoprecipitation with 15 μg of HCAb1 and HCAb2. Immunoprecipitated HSP90 protein was detected on an immunoblot using a commercial anti-HSP90 antibody. HCAb2 pulled down HSP90 from 2 h, 6 h and 24 h tumor lysates while HCAb1 did not pull down HSP90. Equal amounts of HCAb1 and HCAb2 were pulled down as detected by anti-mouse IgG antibody. (TIFF 1627 kb)
Additional file 5: Figure S4. HCAb2-localized xenograft tumor cells express protein disulfide isomerase. A-D. 24 h time point tumor section was incubated with anti-PDI and Alexa Fluor® 488 anti-mouse IgG antibodies. Uniform PDI staining was observed in all cells (panel C) throughout the tumor section. Scale bar represents 10 μm. (TIFF 1344 kb)
Additional file 6: Figure S5. Immunofluorescence detection of CANX and PDI in MDA-MB-231 cells. MDA-MB-231 cells were grown in multi-well chamber slides and incubated with anti-CANX (panel A) or anti-PDI (panel B) antibodies. Uniform CANX and PDI staining was observed in all cells. Scale bar represents 20 μm. (TIFF 3108 kb)
Additional file 7: Figure S6. Calnexin knockdown did not lead to increased HCAb2 binding to MDA-MB-231 cells. A. Immunoblot analysis of MDA-MB-231 cells transiently transfected with mock (transfection medium alone) or luciferase siRNA (siLuc) or calnexin siRNA (siCANX) at different concentrations. β-actin was used as the loading control. B. Flow cytometry analysis of mock, siLuc (25 nM) or siCANX (25 nM) transfected MDA-MB-231 cells using HCAb2. Percentages of cells that showed positive binding with HCAb2 in comparison to isotype control are shown in the histograms. Unstained (blue peak), isotype control (red peak) and HCAb2 (yellow peak). (TIFF 1523 kb)

## Abbreviations

sdAbs: Single domain antibodies
HCAbs: Heavy chain antibodies
HSP90: Heat shock protein 90
CLTC: Clathrin heavy chain
CANX: Calnexin
PDI: Protein disulfide isomerase
CD31: Cluster of differentiation 31
CD44: Cluster of differentiation 44
UPR: Unfolded protein response
Grp94: Glucose-regulated protein 94 kDa
TRAP1: Tumor necrosis factor type 1 receptor-associated protein
cDNA: Complementary DNA
DAPI: 4′,6-Diamidino-2-Phenylindole, Dihydrochloride
SDS: Sodium dodecyl sulfate
PBS: Phosphate buffered saline
EDTA: Ethylenediaminetetraaceticacid
LC-MS: Liquid chromatography mass spectrometry
