# Supplementary figures and images for: Patient-derived heavy chain antibody targets cell surface HSP90 on breast tumors
Source: BMC Cancer. 2015 Sep 3;15:614. doi: 10.1186/s12885-015-1608-z (PMC4559304; doi:10.1186/s12885-015-1608-z)

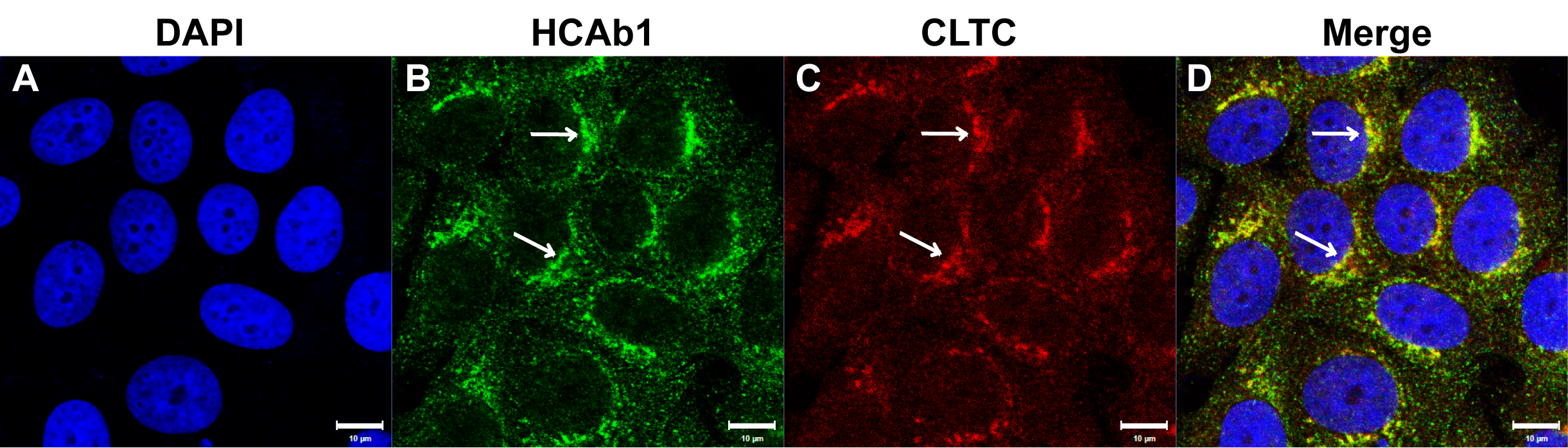

Supplement: Additional file 1: Figure S1. — Clathrin heavy chain protein is the target antigen of HCAb1. A-D. MCF7 cells were incubated with HCAb1 and a commercial anti-clathrin heavy chain antibody. Arrows indicate regions that show co-localization of HCAb1 and anti-clathrin heavy chain antibody. Nuclei were stained with DAPI. Scale bar represents 10 μm. (TIFF 2942 kb) [file 12885_2015_1608_MOESM1_ESM.tiff]

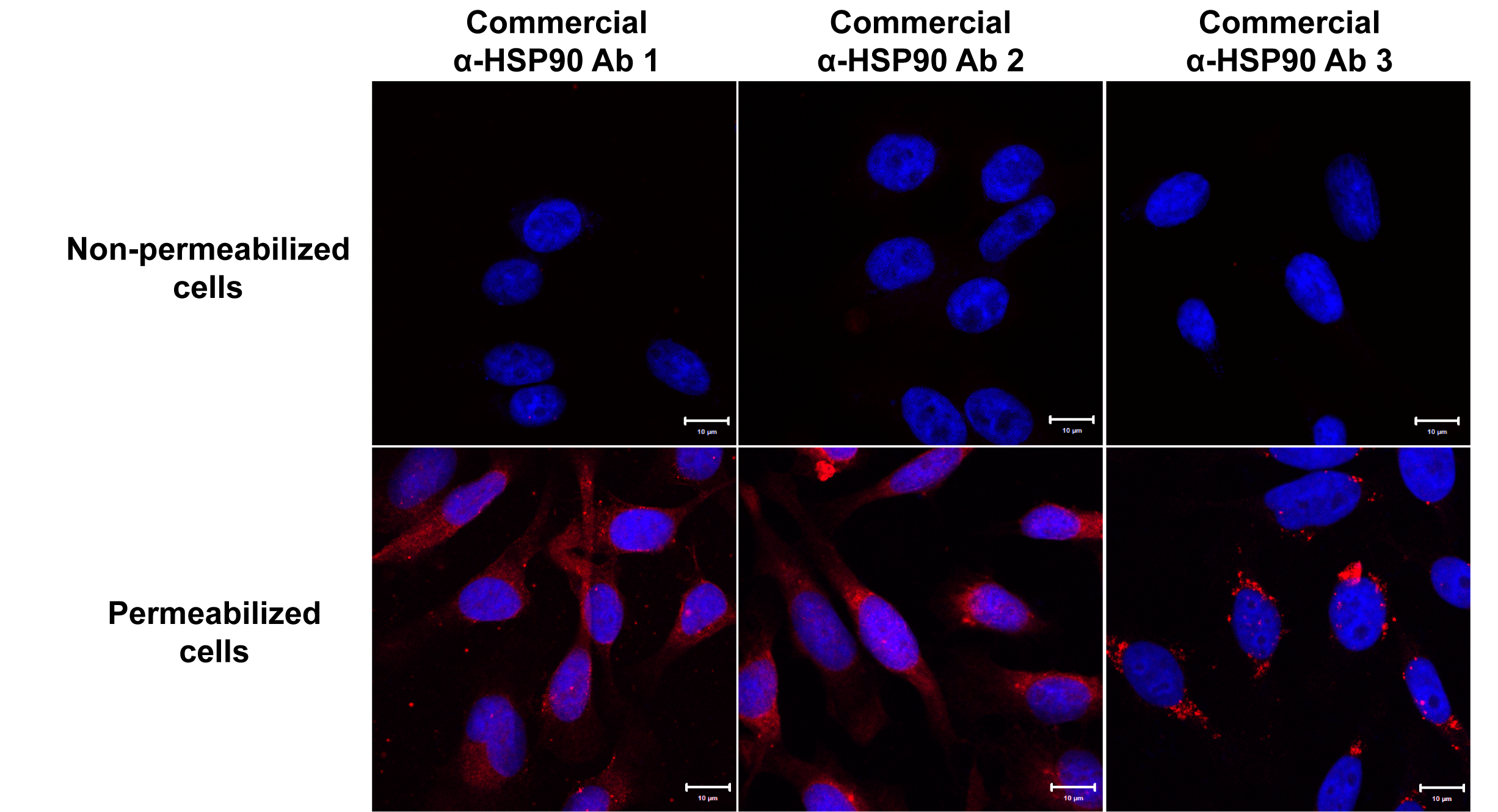

Supplement: Additional file 3: Figure S2. — Commercial anti-HSP90 antibodies did not bind to cell surface HSP90. Immunofluorescence analysis of MDA-MB-231 cells was performed with 3 different commercial anti-HSP90 antibodies. Commercial anti-HSP90 Ab 1 (sc-1055), commercial anti-HSP90 Ab 2 (sc-1057) and commercial anti-HSP90 Ab 3 (CST 4877) were used as indicated. Non-permeabilized cells were used to determine cell surface staining and permeabilized cells were used to determine intracellular staining. Nuclei were stained with DAPI. Scale bar represents 10 μm. (TIFF 2948 kb) [file 12885_2015_1608_MOESM3_ESM.tiff]

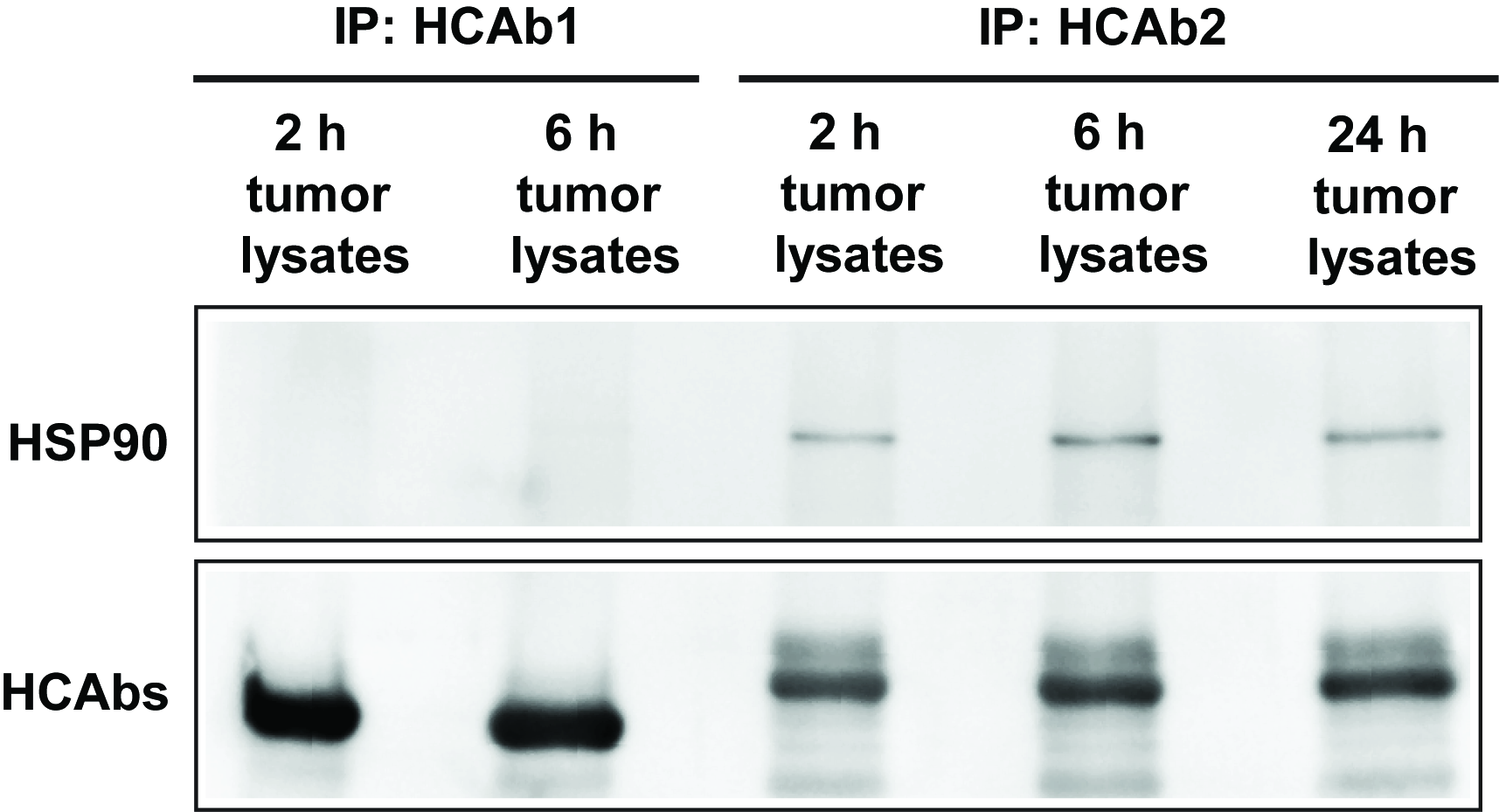

Supplement: Additional file 4: Figure S3. — Immunoprecipitation of HSP90 from xenograft tumor lysates using HCAb2. RIPA lysates of xenograft tumor pieces were used for immunoprecipitation with 15 μg of HCAb1 and HCAb2. Immunoprecipitated HSP90 protein was detected on an immunoblot using a commercial anti-HSP90 antibody. HCAb2 pulled down HSP90 from 2 h, 6 h and 24 h tumor lysates while HCAb1 did not pull down HSP90. Equal amounts of HCAb1 and HCAb2 were pulled down as detected by anti-mouse IgG antibody. (TIFF 1627 kb) [file 12885_2015_1608_MOESM4_ESM.tiff]

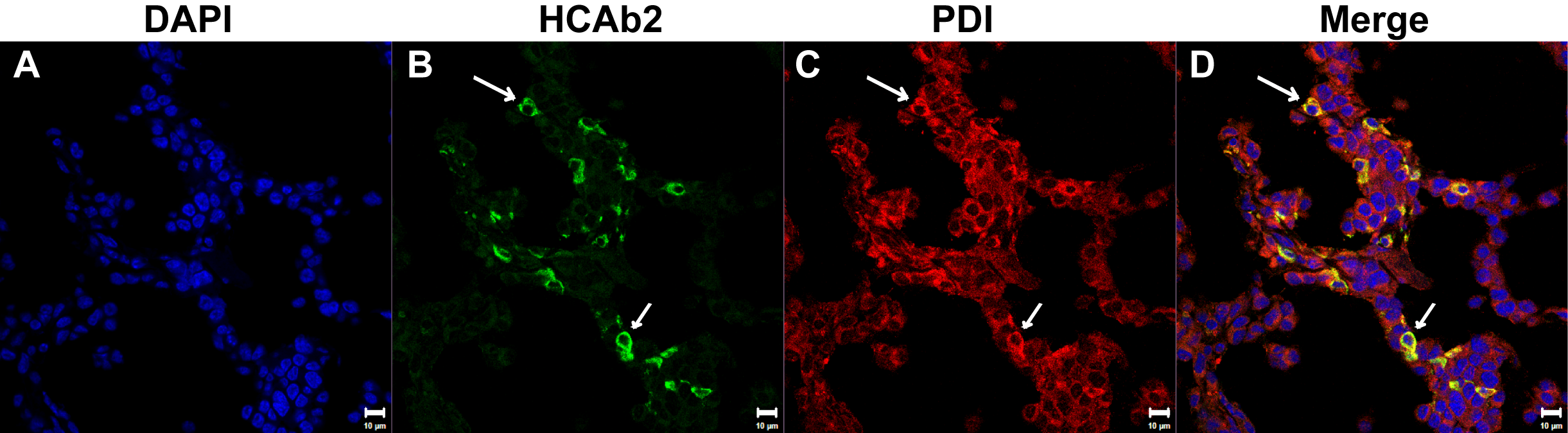

Supplement: Additional file 5: Figure S4. — HCAb2-localized xenograft tumor cells express protein disulfide isomerase. A-D. 24 h time point tumor section was incubated with anti-PDI and Alexa Fluor® 488 anti-mouse IgG antibodies. Uniform PDI staining was observed in all cells (panel C) throughout the tumor section. Scale bar represents 10 μm. (TIFF 1344 kb) [file 12885_2015_1608_MOESM5_ESM.tiff]

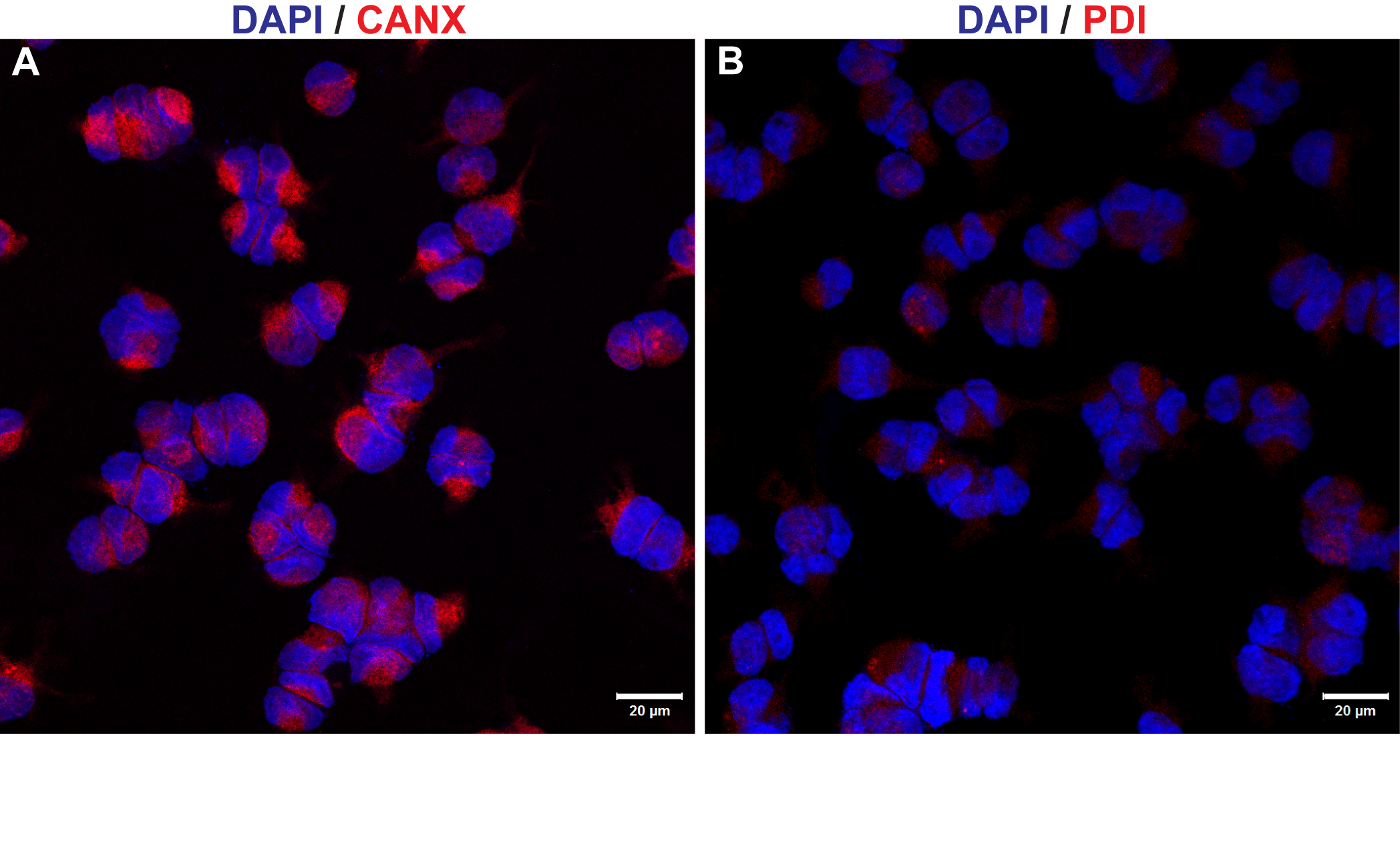

Supplement: Additional file 6: Figure S5. — Immunofluorescence detection of CANX and PDI in MDA-MB-231 cells. MDA-MB-231 cells were grown in multi-well chamber slides and incubated with anti-CANX (panel A) or anti-PDI (panel B) antibodies. Uniform CANX and PDI staining was observed in all cells. Scale bar represents 20 μm. (TIFF 3108 kb) [file 12885_2015_1608_MOESM6_ESM.tiff]

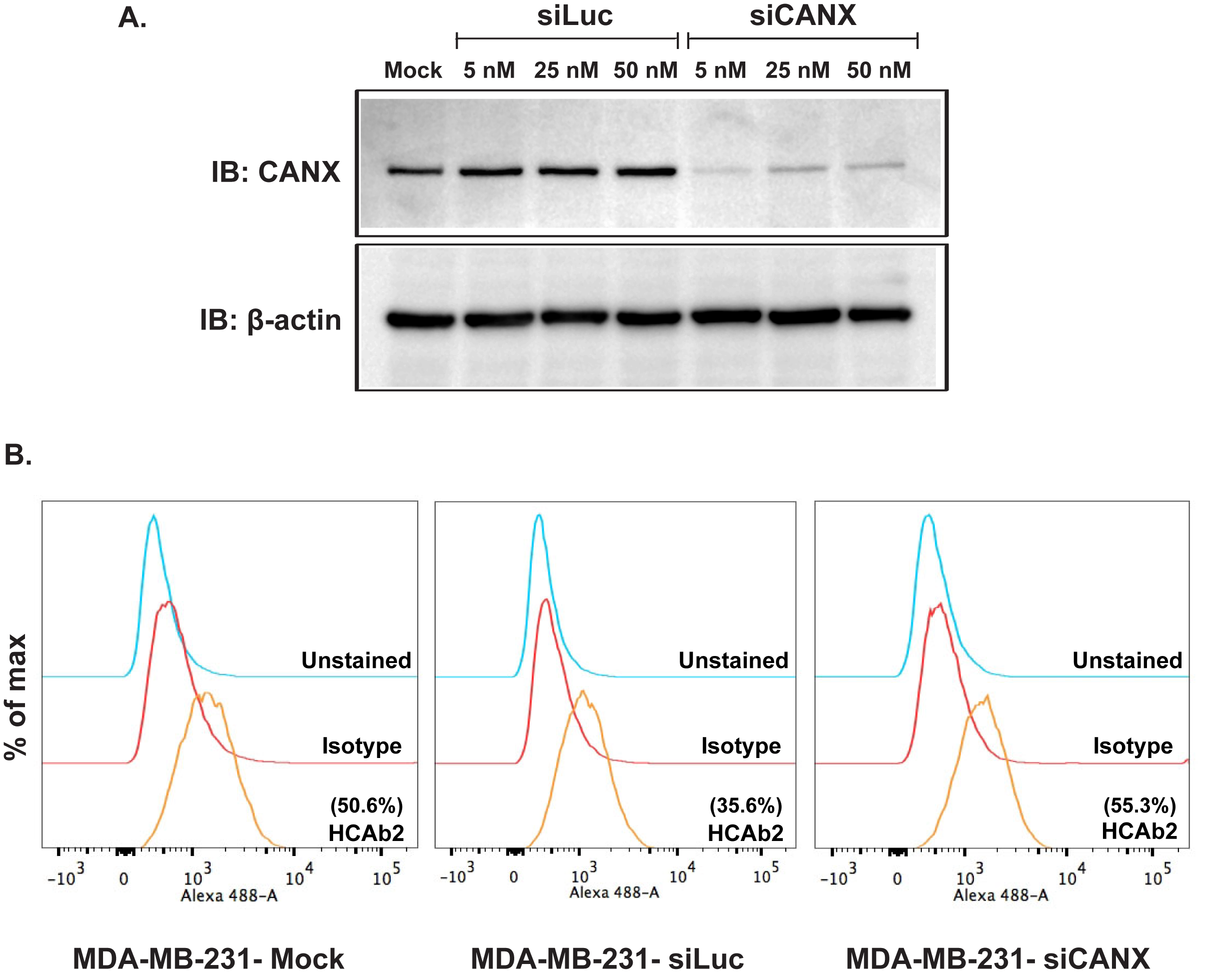

Supplement: Additional file 7: Figure S6. — Calnexin knockdown did not lead to increased HCAb2 binding to MDA-MB-231 cells. A. Immunoblot analysis of MDA-MB-231 cells transiently transfected with mock (transfection medium alone) or luciferase siRNA (siLuc) or calnexin siRNA (siCANX) at different concentrations. β-actin was used as the loading control. B. Flow cytometry analysis of mock, siLuc (25 nM) or siCANX (25 nM) transfected MDA-MB-231 cells using HCAb2. Percentages of cells that showed positive binding with HCAb2 in comparison to isotype control are shown in the histograms. Unstained (blue peak), isotype control (red peak) and HCAb2 (yellow peak). (TIFF 1523 kb) [file 12885_2015_1608_MOESM7_ESM.tiff]
